# Assessment of current prescribing practices using World Health Organization core drug use and complementary indicators in selected rural community pharmacies in Southern India

**DOI:** 10.1186/s40545-016-0074-6

**Published:** 2016-07-19

**Authors:** Anandhasayanam Aravamuthan, Mohanavalli Arputhavanan, Kannan Subramaniam, Sam Johnson Udaya Chander J

**Affiliations:** Department of Pharmacy Practice, JKKMMRFs AJKK SA College of Pharmacy, Vattamalai, Ethirmedu, B Komarapalayam, Namakkal District, 638183 Tamilnadu India; Department of Pharmacy Practice, RVS College of Pharmaceutical Sciences, Coimbatore, 641402 Tamilnadu India

**Keywords:** Prescribing indicators, Patient care indicators, Health facility indicators, WHO guideline, India

## Abstract

**Background:**

Due to the lack of clear, comprehensive, and rational drug policy, the production of pharmaceutical preparations in India is distorted for the most part. Indian markets are flooded with more than 70,000 formulations, compared to approximately 350 formulations listed in the World Health Organization (WHO) Essential Drug List. Studies have indicated that majority of prescriptions in India are of drugs of “doubtful efficacy.” To promote rational drug use in developing countries, assessment of drug use patterns with the WHO drug use indicators is becoming increasingly necessary. The aim of this study was to assess the patterns of drug use by using WHO core drug use and complementary indicators.

**Methods:**

One thousand fifty-two patients were prospectively interviewed and their prescriptions analyzed according to WHO guideline five randomly selected busy community pharmacies in northern district of the State of Tamil Nadu, South India to analyze the WHO core drug use and complementary indicators using an investigator-administered data collection form. The main outcome measured is patterns of drug use measured using WHO core drug use and complementary indicators.

**Results:**

The data obtained showed that, out of total drugs prescribed (3936), only 2.5 % (100) drugs were prescribed by generic name. Mean number of drugs per encounter was 3.7. Use of antibiotics was 22 %, percentage of encounters with an injection was 7.2 %, and the percentage of drugs prescribed from formulary was 99.8 %.

**Conclusions:**

Brand name prescribing is dominated even in rural India. There is a need to improve the availability of essential guidelines and key drugs in the stock in rural areas of India. Prescriptions studied were conforming to most indicators of WHO except the number of drugs prescribed & generic name prescription practice, which deviated. In India the healthcare is dominated by private practitioners at the primary level. Prescription practices of the individual community-based clinician needs consistent monitoring with respect to generic name prescribing habits as well as the number of drugs prescribed. The WHO drug use indicator guidelines need to be promoted amidst the primary care clinicians and should not be just limited to hospitals having a formulary. The data collected by this study can be used by policymakers to monitor and improve the prescribing and consumption of pharmaceutical products in Southern India.

## Background

World Health Organization (WHO) defined rational use of drugs as “patients receive medicines appropriate to their clinical needs, in doses that meet their own individual requirements for an adequate period of time, at the lowest cost to them and their community [1].” Due to the lack of clear, comprehensive, and rational drug policy, the production of pharmaceutical preparations in India is distorted for the most part. Indian markets are flooded with more than 70,000 formulations. Thousands of drug companies operate in India and they manufacture generic preparations with different brand names. Huge number of vitamins, tonics, and multi-drug combinations that are unique to Indian market are also manufactured and marketed here. This entails the drug manufacturers to enter into fierce competition, which makes them to encourage the prescribers to prescribe branded medicines, often in exchange of small favor. This benefits the manufacturing companies, but it also results in drugs being prescribed without any necessity and combinations that are not rational [2, 3]. A concerted effort is needed to readjust pharmaceutical actions and practices aiming at the rational use of medications. WHO developed Medication Use Indicators, including Prescription Indicators with an aim to evaluate the services provided to the population in regards to medications [4]. Prescription Indicators allow the therapeutic actions taken in similar institutions to be ascertained, enabling subsequent comparison of parameters between them, and to evaluate the population’s medication needs and determine the most frequently used medications in a given place [4]. In addition, these indicators enable the investigator to identify the prescription profile and quality of services offered to the population. The prescription Indicators are as follows [4]:

### Average number of drugs per medical prescription

This indicator helps in investigating poly-medication, which is a major factor contributing to adverse drug reactions (ADRs) and drug-drug interactions (DDIs). The educational quality informational level of the prescriber may also be observed [4].

### Percentage of drugs prescribed by generic name

This indicator enables the investigator to calculate the number of prescriptions in which the drugs are prescribed by the generic name. This helps in controlling drug costs in the health service. It also evaluates the marketing influence on the person prescribing drugs [4].

### Percentage of drugs prescribed from essential drug list or formulary

This indicator helps in measuring the degree to which practices conform to the current National Drug Policy (NDP) of October 1998. By following essential drug list, it guarantees the treatment of the principal diseases of the population besides controlling overall cost of medications.

### Percentage of encounters with an antibiotic prescribed

This indicator evaluates the use of antibiotics in excess which contributes to bacterial dissemination and resistance [4].

### Percentage of prescribed injectable drugs

This indicator helps to evaluate the injectables in excess, administration of which may have serious consequences when prescribed or applied wrongly, such as in the event of anaphylactic reactions, adverse reactions, necrosis, etc. [4]. Even though, this indicator helps in evaluation of attention given to health swiftly, these indicators do not quantify all the important aspects of drug use [4].

Studies have indicated that majority of prescriptions in India are of drugs of “doubtful efficacy” [5–7]. Due to high cost of inappropriate use of drugs, developing countries face more problems due to limited economic resources and lack of organized drug policy [8]. To promote rational drug use in developing countries, assessment of drug use patterns with the WHO drug use indicators is becoming increasingly necessary [9, 10].

### The current status of drug use pattern in South India

All evidences available indicate that observations have been made only in hospitals and not in the private clinics that cater to the majority of the population in this part of the country. Even those observations reveal that the prescription pattern is not in line with the WHO drug use guidelines. Though there is a guideline stating that the durgs should be prescribed using the generic names, adherence to that can hardly be observed across this subcontinent [6, 7].

## Aim of the study

To assess the patterns of drug use by using WHO prescribing, health facility, and complementary indicators in selected community pharmacies in Southern India.

## Methods

### Study design

A prospective, cross-sectional survey was carried out in five randomly selected busy community pharmacies from different cities in a northern district of State of Tamil Nadu, South India. The study was conducted between June 2014 and May 2015 in order to consider the prescriptions that flow through all seasonal periods. The study sample comprised of all the medication prescriptions made out of those five selected pharmacies.

### Data collection

Two well-trained clinical pharmacists collected data on prescribing, health facility, and complementary indicators by observing the prescriptions and interviewing the patients. A data collection form prepared exclusively for this study was used to collect data. Information obtained included prescribing date, gender identity, age, and BMI of the patients, educational qualification of patients, physician specialty, prescription-related information such as dosage forms and the number of medicines per prescription, and WHO core drug prescription indicators and complementary indicators. According to a document by WHO “How to investigate drug use in health facilities,” at least 600 prescription encounters should be included in cross-sectional survey to describe the current prescription pattern [10]. The sample selected was by systematic random sampling method. The sampling unit was patient encounters taking place at selected five community pharmacies.

### Prescribing indicators measurement

The WHO prescribing indicators were pretested and slight modification was made to make it applicable in Indian scenario before using in this study. The formula adopted from the WHO’s manual for the assessment prescribing indicators used in this study are as follows:

1. Average number of drugs per encounter = Total number of drugs prescribed/Total number of encounters sampled. (Combinations of drugs prescribed for one health problem were counted as one.
2. Percentage of drugs prescribed by generic name = (Number of drugs prescribed by generic name/Total number of drugs prescribed) × 100.
3. Percentage of encounters with an antibiotic prescribed = (Number of patient encounters with an antibiotic/Total number of encounters sampled) × 100.
4. Percentage of encounters with an injection prescribed = (Number of patient encounters with an injection prescribed/Total number of encounters sampled) × 100.
5. Percentage of drugs prescribed from essential drugs list = (Number of drugs prescribed from essential drugs list/Total number of prescribed drugs) × 100.

### Patient care indicators measurement

Patient care indicators measured were consultation time, dispensing time, and percentage of drugs actually dispensed. Consultation and dispensing times were divided in three categories viz., ≤ 5 min, 6–10 min, and 11–15 min. Percentage of drugs actually dispensed was calculated by dividing the number of drugs actually dispensed at each of the community pharmacy by the total number of drugs prescribed multiplied by 100.

### Health facility indicators measurement

Health facility indicators measured were drugs available in the formulary and drugs not available in formulary. It can be measured by subtracting drugs not available in the formulation from total amount of drugs prescribed × 100.

### Complementary indicators measurement

Complementary indicators analyzed were number of patients treated without drug, number of patient treated with drugs, cost of injections prescribed, and the ratio of drugs prescribed versus drugs dispensed. These were expressed in percentage.

### Data analysis

All data in the data collection form were analyzed using Microsoft Excel 2007. The indicators are reported as means and proportions. From five community pharmacies 218, 215, 197, 220 and 202 prescriptions were analyzed respectively.

## Results

Out of 1052 patient encounters assessed during the study period, 447 (42.5 %) were males and 605 (57.5 %) were females with a mean age of 28.37 years. The classification of patient encounters based on BMI shows that most of the patients fell in the overweight BMI range 25 to 29.9 (35.4 %) followed by normal BMI range of 18.5 to 24.9 (32.8 %). The educational status of the patient encounters reveals that 40.2 % of the patients (*N* = 423) have finished at least undergraduate studies. This is shown in Table 1.

**Table 1:** Socio-demographic characteristics of patients served in five community pharmacies in Southern India

Socio-economic characteristics	Frequency (N)	Percentage (%)
Age (yrs)		
<5 5.1–15 15.1–25 25.1–35 35.1–45 45.1–55 55.1–65 65.1–75 >75.1	110 73 162 429 116 79 67 12 4	10.5 6.9 15.4 40.8 11.0 7.5 6.4 1.1 0.4
Sex		
Male Female	447 605	42.5 57.5
Educational Status		
<SSLC SSLC HSC Diploma UG PG Unknown/Nil	217 89 112 30 423 70 111	20.6 8.5 10.6 2.9 40.2 6.7 10.6
BMI		
<18.5 18.5–24.9 25–29.9 30–34.9 >34.9	75 345 372 198 62	7.1 32.8 35.4 18.8 5.9

*SSLC* secondary school leaving certificate, *HSC* higher secondary certificate, *UG* under graduate, *PG* post graduate

Highest number of prescriptions encountered were from consultant physicians (*N* = 271, 25.8 %) followed by the pediatricians (*N* = 205, 19.5 %), general practitioner (*N* = 180, 17.1 %), gynecologist (*N* = 139, 13.2 %) followed by ENT, orthopedic, and the dentist (*N* = 119, 78, and 41; 11.3, 7.4 and 3.9 % respectively). The dermatologists and the civil surgeons contributed together to 12.5 % of prescriptions in total. The consultant physician was the first choice for the patients as majority of the prescriptions encountered were that of adults, followed by the pediatrician and the general practitioner. The civil surgeon was the last choice for the patients. We could also see from the prescriptions that the non-pediatric patients were also treated by the pediatrician. The indication wise distribution of data shows that up to 40 % of the total prescriptions were given for indications like cough/cold/congestion/fever/headache. Acute infections and the allergic reactions were the major cause for the patients to come to the clinics. This can be shown in Table 2.

**Table 2:** Distribution of physician specialty and indications of prescription encounters

Prescription characteristics	Frequency (N)	Percentage (%)
Physician specialty		
Consulting physician Pediatrician General practitioner Gynecologist ENT Orthopedics Dentist Dermatology Civil surgeon	271 205 180 139 119 78 41 12 7	25.8 19.5 17.1 13.2 11.3 7.4 3.9 1.1 0.7
Indications		
Cough/cold/congestion/fever/headache Back pain & joint pain Gastro Intestinal disorders Injury & pain Bronchospasm/ asthma/ breathing problems Dermatitis Allergy & pain Throat infection Hypertension Giddiness Ear pain Others^a^	422 180 98 69 63 61 54 33 19 16 15 22	40.1 17.1 9.3 6.6 6.0 5.8 5.1 3.1 1.8 1.5 1.4 2.2

^a^Anemia Burns/injury Eye infection Wounds

The distribution of drug prescription indicators shows that more than 53.2 % of the encounters contained four drugs with an average number of drugs per encounter of 3.7, which should always be maintained as low as possible to reduce adverse effect and patient medication cost. Almost 92 % of the prescription encounters contained no drugs prescribed by their generic names and about 58.8 % of prescriptions contained at least one antibiotic and 24.3 % contained at least one injection. Surprisingly, all the drugs dispensed through those 1052 prescriptions were present in essential drug list. The community pharmacies ensure that any prescription is dispensed in full. In the rural areas, when a patient returns with an incompletely filled prescription, he may not return to the pharmacy for any future need as he/she may perceive the pharmacy as one that’s inadequately stocked. At least the community pharmacy owners and the counter managers or the pharmacists ensure that the generic equivalent, whether it is bio-equivalent or not, is substituted for what is prescribed by the physician. So, the availability of drugs from the formulary was 100 %. This can be clearly seen in Table 3.

**Table 3:** Distribution of drug prescription indicators in five community pharmacies in Southern India

Prescribing indicators	Total number of prescriptions (*N* = 1052)	Percentage (%)
Number of drugs per prescription		
One Two Three Four Five	22 79 216 560 175	2.1 7.5 20.5 53.2 16.6
Number of drugs prescribed by generic name		
None One Two	962 80 10	91.4 7.6 1.0
Number of drug encounter with antibiotics		
None One Two	309 619 124	29.4 58.8 11.8
Number of drug encounter with injection		
None One Two	770 256 26	73.2 24.3 2.5
Number of drugs prescribed from essential drug list	1052	100

The patient care indicators consisted of three components such as consultation time, dispensing time and percentage of drug actually dispensed. Maximum number of patients (*N* = 822, 78.1 %), had anywhere between the 6–10 min of consulting time with a physician with an average time of 8 min. The dispensing time required for 82.5 % of the prescription encounters fell between 6 and 10 min range. The dispensing time taken in rural areas was low when compared to another department of other hospital because prescription consists mainly tablets and capsules, which are prescribed in different doses, but at regular intervals. So, pharmacists required less time for the dispensing. In 1052 prescriptions, 3936 drugs were prescribed and the percentage of actual drug dispensed was 99.8 %. This is depicted in Table 4.

**Table 4:** Distribution of patient care indicators in five community pharmacies in Southern India

Patient care indicators	Total number of prescriptions (*N* = 1052)	Percentage (%)
Consultation time (Minutes)		
0–5 6–10 11–15	230 822 0	21.9 78.1 0.0
Dispensing time (Minutes)		
0–5 6–10	184 868	17.5 82.5
Ratio of drugs prescribed/dispensed	3936/3927	99.8 %

A total of 3936 drugs were prescribed from 1052 prescription encounters, in which, 3927 drugs was available in the formulary. 99.8 % of drugs prescribed were dispensed by the community pharmacies. The formulary concept is totally alien to the community pharmacies and is not even followed in many hospitals here. But, for a reference, the prescriptions were catered from the available stock and so we mean the stock of the individual retailer as the formulary – the retailers know about the prescription habits of the physicians in the vicinity and so they could cater to all prescribed brands or its generic substitutes. The data obtained also shows that all patients were treated with drugs and no patient was treated without a drug. We also analyzed injection drug cost per prescription as a complementary indicator. The data obtained shows that most of the injections prescribed (*N* = 195, 80.2 %) fell in the cost range of below Rs. 25. The details of health facility indicator and complementary indicator can be seen in Table 5.

**Table 5:** Health facility indicator results from five community pharmacies in Southern India

Indicators	Frequency	Percentage (%)
Health Facility Indicator (*N* = 3936)		
1. Drugs available in formulary 2. Drugs not available in formulary	3927 9	99.8 0.2
Complementary Indicator (*N* = 1052)		
1. Number of patients treated without drug 2. Number of patient treated with drugs 3. Injectable drug cost (Rs)	0 1052	0.0 100
<25 26–50 51–75	195 40 8	80.2 16.5 3.3
Ratio of drugs dispensed/prescribed	3927/3936	99.8 %

Table 6 shows the details of drugs prescribed out of 1052 prescriptions. A total of 3936 drugs were prescribed from 1052 prescriptions. Antimicrobials agents was most commonly prescribed drugs (*N* = 977, 24.8 %) followed by nutritional supplements (*N* = 483, 12.3 %), anti-ulcer (*N* = 470, 11.9 %) anti-allergic (*N* = 432, 11 %), antipyretic (*N* = 379, 9.6 %), and antacids & LB (*N* = 298, 7.6 %).

**Table 6:** Drug class prescribed in five community pharmacies in Southern India

Drug class	Frequency (*N* = 3936)	Percentage (%)
Antibacterial	977	24.8
Nutritional Supplements, Vitamins, etc./Saline	483	12.3
Antiulcer	470	11.9
Antiallergic	432	11.0
Antipyretics	379	9.6
Antacid & LB	298	7.6
Antiinflammatory	229	5.8
Analgesics	121	3.1
Anti-diabetics	75	1.9
Anti-cold	73	1.9
Anxiolytics	64	1.6
Steroids	54	1.4
Nasal Decongestants	53	1.3
Anti-vertigo	52	1.3
CVS	45	1.1
Others^a^Topicals	131	3.5

^a^Topical, CNS, Anti-emetic, Anti-amoebic, Laxative, Anti-diarrheal, Urinary Alkalizer, Musculoskeletal, Enzymes, Antispasmodic drugs

Antibiotics are used both as single and as fixed dose combination and prescribed orally. NSAIDS are also prescribed in tablet form in double dose combination.

### Limitations of the study

- Only 1052 prescription encounters were sampled for this study and a higher number of prescription encounters are required for better result. This cannot be generalized to overall prescribing practice of all private practitioners.
- Prescribing indicators based drug utilization study in health facilities is not an exhaustive tool to identify all problems related to prescribing and rationality of the drug use, as they do not exactly explain why drugs are prescribed.
- The WHO prescribing indicators measure aspects of outpatient treatment. They are designed for use in health centers, dispensaries or hospital outpatient departments. The prescribing indicators are less useful in specialty outpatient clinics in referral hospitals where the drug use pattern is more complex.

## Discussion

In this study, 91.4 % prescriptions did not contain even a single drug prescribed in its generic name. A similar study conducted in Goa, the western part of India has similar observations where only 0.05 % of the drugs out of 1842 products prescribed were in generic name [10]. This study showed that, in the community pharmacies, where this study was conducted, most of the indicators analyzed were in agreement with the WHO’s recommendations, except for the mean number of drugs per encounter and percentage of medications prescribed by generic name which should be ≤2 and 100 %, respectively [4, 9]. Fixed dose combinations (FDCs) are one of the strategies to reduce the number of drugs prescribed and improve medication compliance. Increasing requirement of drugs in patients with more than one disease justifies the use of FDCs [11, 12]. But with the recent ban on 344 FDCs, a question arises on how many prescribed during the study were rational? A similar study highlights the practice of poly-pharmacy, low usage of generic drugs, injudicious usage of antibiotics and injections and low usage of drugs from essential drugs list in other parts of this region in India [13].

## Conclusion

The study indicates a clear need for a guideline by the policy making bodies directing the prescription habits of all clinicians in this part of India. The rules are defined and available even today, but the practice is yet to change is the sad part of the story. Unless strict enforcement of the guidelines happen with consistent monitoring, adherence by clinicians leading to better healthcare services cannot be guaranteed in this part of India.
